# Systemic arterial air embolism following computed tomography (CT)-guided percutaneous lung biopsy: Case series and review of underlying risk factors, treatment and preventive strategies

**DOI:** 10.1016/j.clinme.2025.100530

**Published:** 2025-11-17

**Authors:** Maimuna Adamu, Chris Skillicorn, Timothy Stone, Harmesh Moudgil, Chulangani Abayaratne

**Affiliations:** a Specialty Registrar, Department of Respiratory Medicine, Princess Royal Hospital Telford, Shrewsbury and Telford Hospital NHS Trust, Telford, United Kingdom; b Consultant Radiologist, Department of Radiology, Princess Royal Hospital Telford, Shrewsbury and Telford Hospital NHS Trust, Telford, United Kingdom; c Consultant Physician, Department of Respiratory Medicine, Princess Royal Hospital Telford, Shrewsbury and Telford Hospital NHS Trust, Telford, United Kingdom

**Keywords:** Air embolism, Lung biopsy, Pneumocephalus, Lung cancer

## Abstract

**Background:**

Systemic arterial air embolism is an uncommon but potentially fatal complication of CT-guided transthoracic lung biopsy. Although rare, it carries significant morbidity due to cerebral and coronary ischaemia.

**Case presentation:**

We report two cases of systemic arterial air embolism occurring during CT-guided percutaneous lung biopsies. Both patients developed acute neurological deficits immediately following the procedure, attributed to cerebral air embolism. Imaging confirmed the presence of intracranial air in subarachnoid spaces. Neurological symptoms improved with supportive management, including oxygen administration and positioning. Histopathology in both cases revealed pulmonary adenocarcinoma.

**Conclusion:**

These cases highlight the importance of prompt recognition and appropriate management of systemic air embolism. Risk mitigation strategies include careful patient positioning, minimisation of intrapulmonary pressure gradients, and maintenance of needle occlusion. Although rare, this complication must be anticipated by interventionalists and managed promptly to avoid permanent neurological damage.

## Introduction

While generally safe, CT-guided percutaneous transthoracic lung biopsy risks pneumothorax, haemorrhage and, rarely, systemic air embolism, where incidence ranges from 0.02% to 0.07%^1^^,^^2^ but with some retrospective studies suggesting a higher figure^3^ with under-recognition of asymptomatic cases.^1^^,^^3^^,^^4^ We present two cases and discuss the underlying mechanisms, risk factors and preventive strategies.

## Case 1

**Fig. 1:**
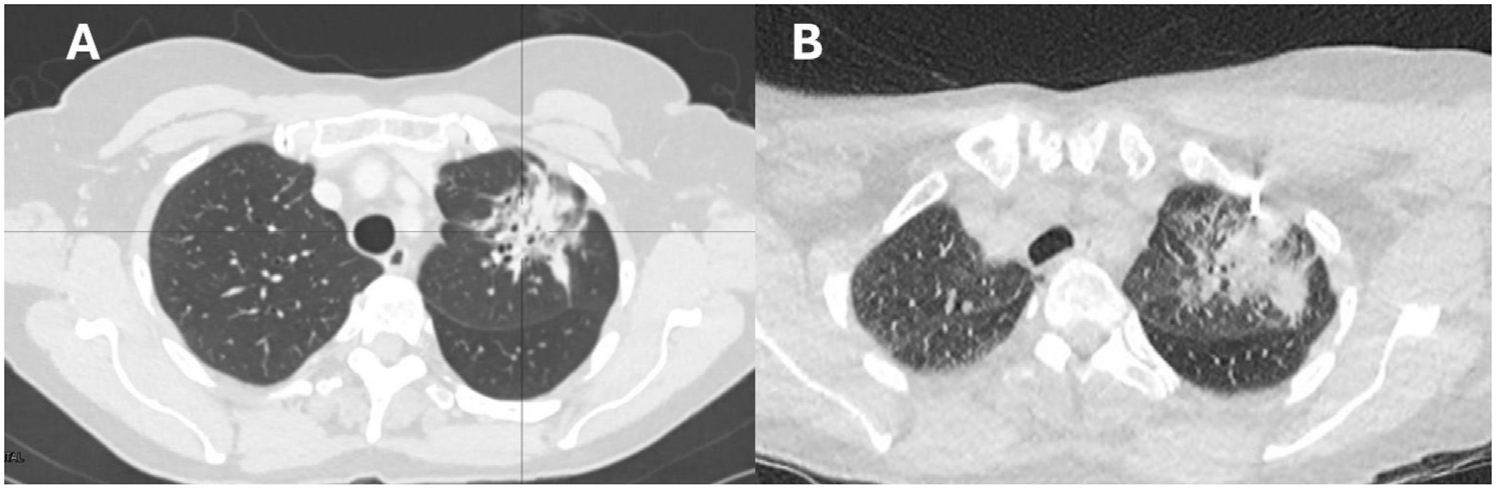
CT scan lung view (A) left upper lobe consolidation (B) biopsy needle.

A 71-year-old woman presented with a non-resolving left upper lobe consolidation (Fig. 1) for which histopathologic confirmation was sought. With the patient supine and under intermittent CT guidance, two passes were made using an 11 cm/18G coaxial core biopsy needle (Fig. 1) targeting the lesion peripherally avoiding central bronchiectatic changes.

Following the second pass, the patient coughed, and the needle was withdrawn using a water-seal technique to minimise air ingress. Immediate post-procedural CT revealed a small pneumothorax and minor parenchymal haemorrhage. Five minutes later, the patient developed acute left hemiparesis and facial weakness. She remained alert, with stable vital signs, and had minor haemoptysis. She was positioned in the right lateral decubitus position and received 15 L oxygen via non-rebreathe mask. The pneumothorax did not require drainage.

**Fig. 2:**
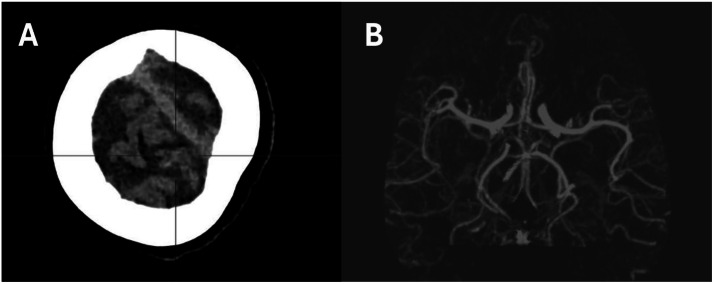
Images of the brain post-biopsy (A) CT scan showing pneumocephalus, (B) CT intracranial angiogram.

**Fig. 3:**
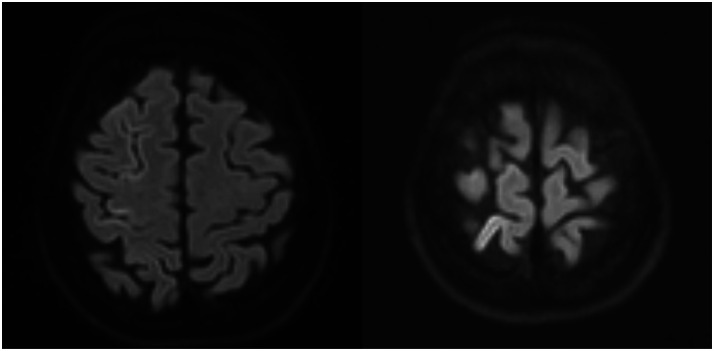
MRI head.

CT revealed air locules in the subarachnoid space overlying the cerebral convexities confirming cerebral arterial air embolism (Fig. 2) but no air in the cardiac chambers, great vessels or thoracic aorta. Subsequent CT angiogram of Circle of Willis showed complete resolution of air bubbles. The following day, MRI of the brain demonstrated no infarction but mild cortical signal changes suggestive of transient ischaemia (Fig. 3). Only mild residual ataxia persisted after 24 h. Histopathology revealed pulmonary adenocarcinoma.

## Case 2

**Fig. 4:**
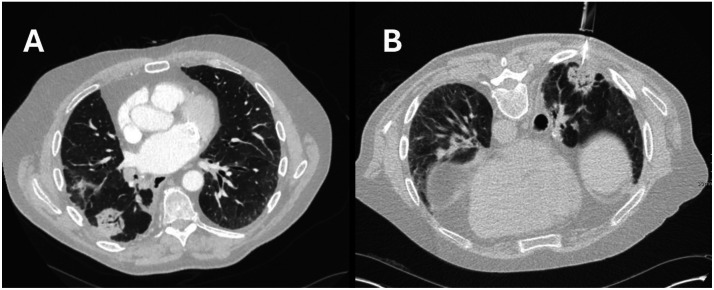
CT scan lung view (A) right middle lobe consolidation (B) biopsy needle.

A 71-year-old man, with a previous right lower lobectomy, underwent CT-guided biopsy of new right middle lobe non-resolving consolidation (Fig. 4). Positioned prone, a single pass using a 6 cm 18-guage needle was performed but shortly after, on attempting to roll the patient onto a trolley, he suffered a self-terminating generalised tonic-clonic seizure. His initial vital signs were stable with saturation of 100% on 15 L oxygen via non-rebreathe mask.

**Fig. 5:**
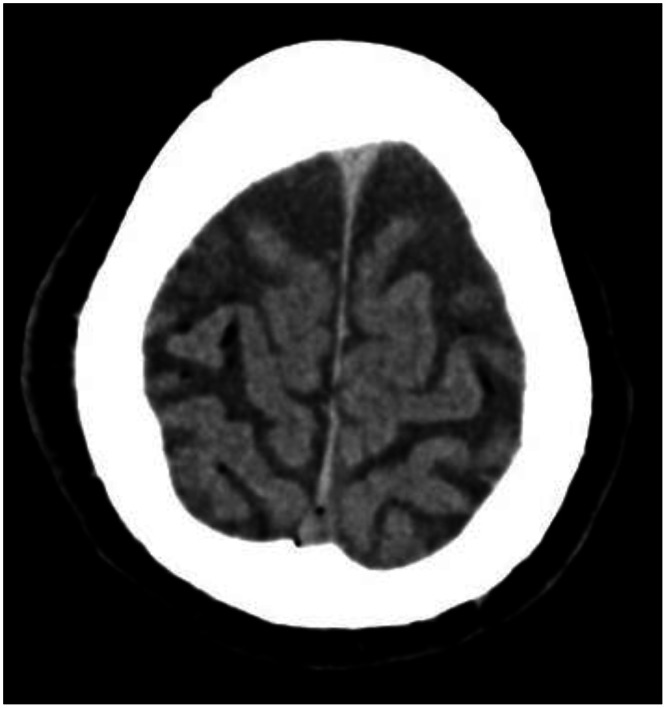
CT scan head pneumocephalus.

**Fig. 6:**
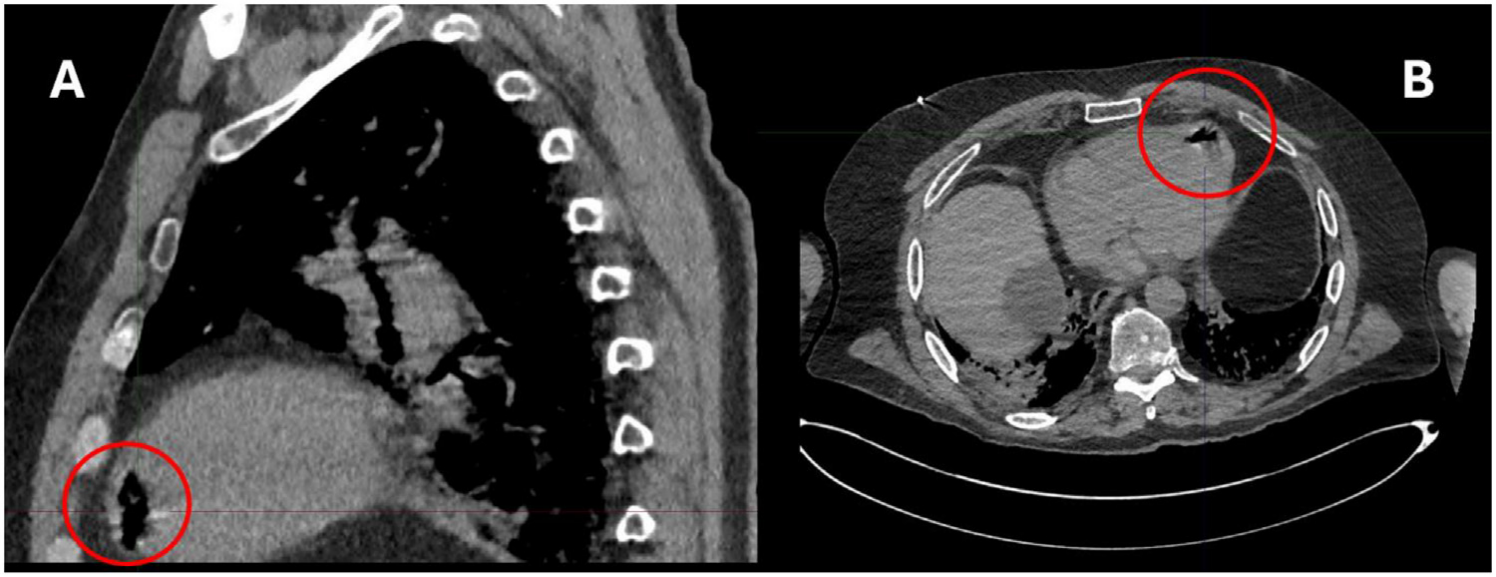
CT scan post-biopsy soft tissue view intraventricular air. (A) sagittal view (B) axial view.

CT imaging revealed small air bubbles within intracranial vessels (Fig. 5) and within the left ventricle (Fig. 6). He regained full consciousness the following day but had residual left-sided weakness. Repeat CT scan showed a reduction in the volume of intracranial gas. He underwent rehabilitation but had some residual left-sided paresis. Histology confirmed pulmonary adenocarcinoma.

## Discussion

CT-guided percutaneous transthoracic lung biopsy (PTLB) investigating indeterminate pulmonary lesions has become an essential diagnostic tool for which complications of systemic arterial air embolism (SAAE) are exceedingly rare;^3^ acute neurological presentations include altered mental status, hemiparesis, aphasia, seizures, or coma due to cerebral arterial embolisation.^3^ Arrhythmias, hypotension, myocardial ischaemia or cardiac arrest may occur when emboli involve coronary arteries.^3^ The volume of air required to induce clinically significant cerebral ischaemia is minimal; as little as 2 mL can be fatal, and even 0.5–1.0 mL air in the coronary arteries may precipitate cardiac arrest.^1^^,^^3^

SAAE results from the inadvertent introduction of atmospheric air into the pulmonary venous circulation with embolisation systemically. Three pathophysiological mechanisms have been proposed: the first involves direct air entry into the pulmonary vein when the biopsy needle stylet is removed, particularly if the patient inhales deeply or coughs, leading to a negative pressure gradient favouring air ingress;^1^^,^^3^^,^^4^ the second, and most probable in both cases presented, is a transient bronchovenous fistula^1^, ^2^, ^3^, ^4^ when the biopsy needle connects an air-containing structure (eg patent bronchi or alveoli) with an adjacent pulmonary vein, establishing a conduit for air embolisation under elevated intrathoracic pressure and where coughing, valsalva or straining can drive air into the systemic circulation: the third, more theoretical, involves the passage of air from the pulmonary arterial system through the capillary bed into the pulmonary veins.^1^, ^2^, ^3^, ^4^

The risk of SAAE is multifactorial. Critical is where the lesion is vertically above the left atrium;^1^, ^2^, ^3^, ^4^ lying prone, these lesions are at reduced venous pressure and the gradient increases the likelihood of air entrainment into the pulmonary veins during phases of negative intrathoracic pressure or abrupt pressure fluctuations as with coughing.^1^, ^2^, ^3^, ^4^ The prone position itself may not be an independent risk factor and rather it is the resultant anatomical relationship of the lesion to the left atrium.^1^, ^2^, ^3^, ^4^ Conversely, the ipsilateral dependent position places the lesion below the left atrial plane and reduces the rate of systemic air embolism.^2^ The frequency of sampling, the angle of needle insertion and trajectory as well as depth of penetration are additional procedural risks,^1^, ^2^, ^3^, ^4^ although not all conclusive as independent risk predictors; deeper needle penetration into the lesion increases the probability of transgressing both airways and pulmonary veins, potentially creating bronchovenous fistula formation.

CT imaging of the brain and thorax remain pivotal to diagnosis often revealing intracranial air within subarachnoid spaces or sulci, and occasionally air in the left cardiac chambers, aorta, or other systemic arteries. Management is primarily supportive. The biopsy needle should be removed using a water-seal technique to prevent further air entry.^3^, ^4^, ^5^ Right lateral decubitus position of the patient can trap intraventricular air in non-dependent regions of the heart and reduce the risk of embolisation.^1^, ^2^, ^3^, ^4^ Administration of 100% oxygen is recommended,^1^^,^^3^^,^^4^ as this accelerates nitrogen washout reducing bubble volume and improves oxygenation of ischaemic tissues.^4^ Occasionally, hyperbaric oxygen therapy is the used enhancing oxygen delivery to hypoxic tissues and resorption of intravascular air.^2^^,^^4^

In summary, SAAE is a rare but serious complication of CT-guided lung biopsy where a high index of suspicion, early imaging and supportive management are paramount to ensure patient safety. Risks may be mitigated with procedural planning with attention to positioning the patient relative to the lesion, the needle path, minimising sampling and cough suppression during biopsy.^2^, ^3^, ^4^, ^5^

## Funding

This research did not receive any specific grant from funding agencies in the public, commercial or not-for-profit sectors.

## Consent for publication

Written informed consent for publication of clinical details and images was obtained from the patient in Case 1. Written informed consent was for publication of clinical details and images was obtained from the daughter of the patient in Case 2, as he is deceased.

## CRediT authorship contribution statement

**Maimuna Adamu:** Writing – original draft, Visualization, Resources, Conceptualization. **Chris Skillicorn:** Writing – review & editing. **Timothy Stone:** Resources, Conceptualization. **Harmesh Moudgil:** Writing – review & editing, Supervision. **Chulangani Abayaratne:** Writing – review & editing, Supervision, Resources, Conceptualization.

## Declaration of competing interest

The authors declare that they have no known competing financial interests or personal relationships that could have appeared to influence the work reported in this paper.
